# Resilience of Metabolically Active Biofilms of a Desert Cyanobacterium Capable of Far-Red Photosynthesis Under Mars-like Conditions

**DOI:** 10.3390/life15040622

**Published:** 2025-04-07

**Authors:** Giorgia Di Stefano, Mickael Baqué, Stephen Garland, Andreas Lorek, Jean-Pierre de Vera, Manuele Ettore Michel Gangi, Micol Bellucci, Daniela Billi

**Affiliations:** 1 Department of Biology, University of Rome Tor Vergata, 00133 Rome, Italy; giorgia.distefano17@gmail.com; 2 PhD Program in Cellular and Molecular Biology, Department of Biology, University of Rome Tor Vergata, 00133 Rome, Italy; 3 German Aerospace Center (DLR), Institute of Planetary Research, Department of Planetary Laboratories, 12489 Berlin, Germany; mickael.baque@dlr.de (M.B.); stephen.garland@dlr.de (S.G.); andreas.lorek@dlr.de (A.L.); 4 German Aerospace Center (DLR), Space Operations and Astronaut Training, Microgravity User Support Center (MUSC), 51147 Köln, Germany; jean-pierre.devera@dlr.de; 5 Italian Space Agency, 00133 Rome, Italy; manuele.gangi@asi.it (M.E.M.G.); micol.bellucci@asi.it (M.B.)

**Keywords:** Mars simulation, desert cyanobacteria, habitability

## Abstract

The response of the desert cyanobacterium *Chroococcidiopsis* sp. CCMEE 010 was tested in Mars simulations to investigate the possibility of photosynthesis in near-surface protected niches. This cyanobacterium colonizes lithic niches enriched in far-red light (FRL) and depleted in visible light (VL) and is capable of far-red light photoacclimation (FaRLiP). Biofilms were grown under FRL and VL and exposed in a hydrated state to a low-pressure atmosphere, variable humidity, and UV irradiation, as occur on the Martian surface. VL biofilms showed a maximum quantum efficiency that dropped after 1 h, whereas a slow reduction occurred in FRL biofilms up to undetectable after 8 h, indicating that UV irradiation was the primary cause of photoinhibition. Post-exposure analyses showed that VL and FRL biofilms were dehydrated, suggesting that they entered a dried, dormant state and that top-layer cells shielded bottom-layer cells from UV radiation. After Mars simulations, the survivors (12% in VL biofilms and few cells in FRL biofilms) suggested that, during the evolution of Mars habitability, near-surface niches could have been colonized by phototrophs utilizing low-energy light. The biofilm UV resistance suggests that, during the loss of surface habitability on Mars, microbial life-forms might have survived surface conditions by taking refuge in near-surface protected niches.

## 1. Introduction

Evidence widely supports that the Martian surface once had considerable liquid water and nutrient sources to sustain autotrophic metabolism [1]. Similar to the Noachian Mars [2,3], the Archean Earth lacked an ozone layer, enabling low-wavelength (200–315 nm) UV irradiance to reach its surface [4,5]. Therefore, it is possible that early Mars might have supported life, although its surface is now inhospitable due to the absence of liquid water, low atmospheric pressure, extreme low temperatures, and high levels of ionizing and UV radiation [6].

On Earth, the increasing dryness of deserts fosters a shift from edaphic to endolithic microbial communities; therefore, it is conceivable that, if life ever occurred on Mars, it may have retreated to subsurface environments as surface habitability was lost [7]. Although there is no general agreement on whether photosynthesis ever occurred on Mars [8,9], the discovery of cyanobacteria capable of using wavelengths beyond visible light (VL) and thriving in shadow environments, including even caves [10], offers a new perspective on near-surface habitability on Mars.

Far-red light photoacclimation (FaRLiP) is an ecologically important process on Earth that allows some cyanobacteria to utilize far-red light (FRL) for oxygenic photosynthesis in environments with attenuated VL and enriched FRL due to physical conditions or the presence of competing photosynthetic organisms [11]. This process includes the replacement of photosynthetic proteins and the synthesis and incorporation of about 8% far-red shifted chl *f* into the photosystem [12]. The possibility of far-red photosynthesis under non-Earth conditions has implications for the habitability of exoplanets orbiting M-class stars that are characterized by a light spectrum peaking in the far-red and infrared, which would select for organisms capable of utilizing near-infrared light [13]. The feasibility of oxygenic photosynthesis under M-dwarf light is supported by the reported capability of cyanobacteria and more complex photosynthetic organisms to grow and produce oxygen under laboratory simulations of M-dwarf light [14,15]. Furthermore, putative far-red acclimated phototrophs could result in a shift of the “red edge” with implications for biosignaure detection since the photosynthetic pigment reflectance is considered a remote indication of life [16].

Since extreme deserts are considered Mars analog fields, desert cyanobacteria provide a unique model for understanding the habitability of near-surface niches on Mars [17]. A few FaRLiP strains belonging to the genus *Chroococcidiopsis* have been identified among isolates from extreme deserts [18], where they colonize lithic substrates characterized by FRL-shifted transmission spectra [19,20].

In view of their astrobiological relevance, desert *Chroococcidiopsis* strains were subjected to space and Mars-like simulations using the EXPOSE facility installed outside the International Space Station [21]. Dried cells survived mainly due to UV radiation shielding provided by top-layer cells in thick biofilms and to regolith in thin cell layers mixed with Martian soil simulants [22,23]. Nevertheless, desert *Chroococcidiopsis* strains have not been exposed to Mars-like conditions in a metabolically active state to date.

In this context, the response of metabolically active biofilms to Mars-like conditions was explored, using the desert cyanobacterium *Chroococcidiopsis* sp. CCMEE 010, which is capable of FaRLiP [24]. Hydrated biofilms were exposed for 3 days using the Mars Simulation Chamber at the Planetary Analogue Simulation Laboratory (PASLAB, DLR Berlin). The effect of a low-pressure atmosphere, variable humidity, and UV irradiation on photosystem II (PSII) activity was evaluated through the in-situ monitoring of Chl *a* fluorescence. After 3 days of exposure, the photosynthetic pigments in the biofilms were analyzed by evaluating the permanence of autofluorescence signals, and viability was assessed using a test based on esterase activity.

## 2. Materials and Methods

### 2.1. Cyanobacterial Strain, Growth Conditions, and Biofilm Development

The cyanobacterial strain *Chroococcidiopsis* sp. CCMEE 010 was isolated from an endolithic community in a rock sample collected in the Negev Desert and it is part of the Culture Collection of Microorganisms from Extreme Environments (CCMEE) established by E. Imre Friedmann and Roseli Ocampo-Friedmann and currently maintained at the Department of Biology, University of Tor Vergata.

A set of twelve biofilms were obtained by plating about 5 × 10^8^ cells on the top of BG-11 agarized medium in Petri dishes sealed with Parafilm as previously reported [23]. A set of six biofilms (four for Mars simulations and two controls) were grown for one month at 25 °C under a constant photon flux density of 10 µmol/m^2^/s, which was provided with a white LED light (4000 K, OSRAM, MI, Italy). Other six biofilms (four for Mars simulations and two controls) were grown for one month at 25 °C under FRL of 40 µmol/m^2^/s provided with a 750 nm LED (L750-01AU Epitex, Ushio GmbH, Germany). Ushio Germany GmbH

### 2.2. Mars Simulation Chamber

**Figure 1:**
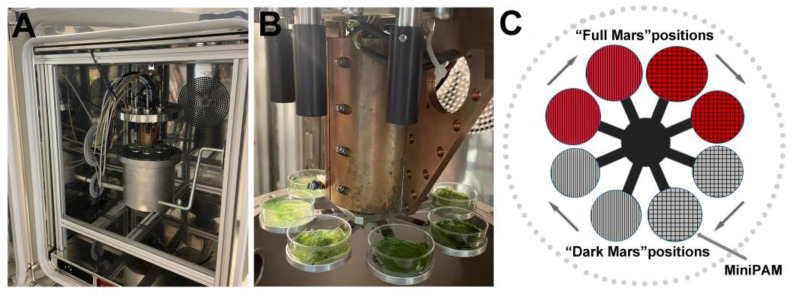
Mars simulation chamber at PASLAB—DLR Berlin (**A**) and a detail of the experiment chamber with the samples in holders (**B**). A graphical representation of the rotating platform, showing the sample distribution in the sample holders (**C**).

Experiments were carried out using the Mars Simulation Chamber at the Planetary Analogue Simulation Laboratory (PASLAB, DLR Berlin) [25], which contains an Experiment Chamber consisting of a vacuum-sealed stainless-steel vessel with an interior rotating platform with eight round aluminum sample holders (Figure 1A). Four holders, designated as “Full Mars”, were UV-irradiated with a 150 W Xe-UV lamp that simulated the complete Martian solar spectrum (200 nm to 2200 nm) on a 13 mm diameter spot using adjustable lenses (Figure 1B,C). The UV radiation dose was measured with an X92-optometer and an RCH-106-4 probe (Gigahertz-Optik GmbH, Germany) at wavelengths ranging from 250 to 400 nm [26]. The UV lamp was active for 16 h and switched off for 8 h daily to simulate the mid-latitude summer Sun’s diurnal cycle for a total of 48 h of irradiation. Four holders, designated as “Dark Mars”, were maintained in dark conditions [25,26] (Figure 1B,C). The Experiment Chamber is equipped with electrical connectors, gas inlet/outlet connectors, four optical fibers for UV light transmission, and one fiber for photosynthetic activity measurements using a photosynthesis yield analyzer (Mini PAM, Walz GmbH, Germany). Two SHT75 sensors (Sensirion AG, Switzerland) integrated with two Pt-100 temperature sensors are installed inside the chamber to monitor humidity and temperature near the turntable [27]. The humidity sensors were calibrated for the Martian atmosphere [27]. Gas flow was regulated by a gas-mixing system capable of controlling up to five gases, with the precise regulation of gas humidity achieved using a CO_2_ humidification system. Internal pressure was managed through a membrane vacuum pump (MV10Vario, Vacuubrand GmbH) [25]. The entire system was controlled by a DAQ-system device (National Instruments Corp., USA) for controlling the time profiles (e.g., simulated daily variations) of the different parameters (e.g., temperature, humidity, irradiation, and atmospheric pressure and composition) in conjunction with the LabView software (National Instruments, Austin, TX, USA).

During the period of exposure, two VL and two FRL biofilms were kept in an incubator under VL and FRL conditions in a laboratory in Rome for controls.

### 2.3. Biofilm Integration in the Mars Simulation Chamber

A total of eight biofilms of *Chroococcidiopsis* sp. CCMEE 010, four of which were grown under VL conditions and four of which were grown under FRL, were placed in the eight sample holders of the Experiment Chamber. In particular, two VL and two FRL biofilms were allocated to the “Full Mars” positions and two VL and two FRL biofilms were allocated to the “Dark Mars” positions (Figure 1C).

The Experiment Chamber conditions were set to simulate the thermo-physical conditions typical of Mars at mid/low latitudes. During a complete run, a dry gas mixture containing 95% CO_2_ and 5% air (4% N_2_ and 1% O_2_) was continuously supplied to the chamber. The pressure was approximately 600 Pa, and the UV lamp was active for 16 h and switched off for 8 h daily to simulate the mid-latitude summer of the solar diurnal cycle (Table 1).

### 2.4. Photosynthetic Activity Measurement

During the 3-day simulation, the photosynthetic activity of the *Chroococcidiopsis* sp. CCMEE 010 biofilms was evaluated every 30 min by measuring the Chl *a* fluorescence [27] with a pulse–amplitude–modulation (MiniPAM) photosynthesis yield analyzer interfaced with the Mars Simulation Chamber. The MiniPAM device applies a 1 s saturating light pulse with an excitation of 5000 μmol m^−2^ s^−1^ of red light (650 nm) through a light fiber located at about 1.5 cm from the sample. This saturation pulse causes the electron transport chain to fully reduce, opening the PSII reaction centers, suppressing photochemical activity, and allowing Chl *a* to emit a fluorescent signal in response to de-excitation. Fluorescence measurements were expressed as the PSII quantum yield (Y) through the following equations: (i) Y(II) = (F_M_′ − F)/F_M_′ for light-adapted cyanobacteria and (ii) F_V_/F_M_ = (F_M_ − F_0_)/F_M_ for dark-adapted cyanobacteria, where F (light-adapted cyanobacteria) and F_V_ (dark-adapted cyanobacteria) are the fluorescence values measured before the MiniPAM saturation pulse triggering; F_M_ is the maximum fluorescence measured after dark adaptation; F_0_ is the minimum fluorescence yield; and F_M_’ is the maximum fluorescence reached during the saturation pulse, measured in light conditions when PSII reaction centers are open [27,28].

### 2.5. Photosynthetic Pigment Autofluorescence

The photosynthetic pigment autofluorescence of the *Chroococcidiopsis* sp. CCMEE 010 biofilms was assessed using a fluorescence microscope (Olympus BX61 Fluorescence Microscope, Olympus Corp., Japan) interfaced with an X-Cite 120Q device (Lumen Dynamics, Canada). A TRITC filter with an excitation wavelength of 480 ± 40 nm and an emission wavelength of >510 nm was used to detect chlorophyll and phycobiliprotein fluorescence signals. Slides were prepared using biofilm samples of about 1 mm^2^, which were observed with a 40× objective lens.

### 2.6. Cell Viability

The biofilms (about 1 mm^2^) were resuspended in BG-11 medium, allowed to rehydrate for 2 h, and stained with calcein acetoxymethyl (calcein-AM) dye (Thermo Fisher Scientific Inc., Waltham, MA, USA). The cells were gently harvested from the biofilms, washed three times, resuspended in 500 µL of BG-11 containing 10 µL of Calcein-AM (1 mg/mL in dimethylsulfoxide), and incubated in the dark at room temperature for 90 min, as previously described [29]. Then, cells were then washed three times with PBS (1×) and resuspended in 10 µL of BG-11 containing 1.5% (*w*/*v*) agarose on top of microscope slides and examined with a Confocal Laser Scanning Microscopy System (CLSM; Olympus Fluoview 1000). Images were acquired using a 60× objective lens and processed using the Imaris v. 6.1.0 software (Bitplane AG Zürcher, Switzerland). The autofluorescence of photosynthetic pigments (chlorophylls) was revealed by exciting the samples with a 635 nm laser; for the calcein fluorescence, a 488 nm laser was used as an excitation source.

## 3. Results

### 3.1. Experimental Mars-like Conditions

**Figure 2:**
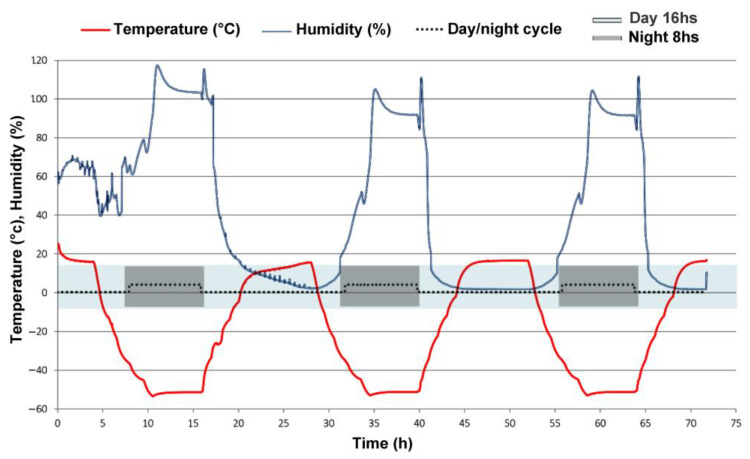
Representation of 3 days of Mars-like diurnal cycle of relative humidity (blue line) and temperature (red line) under “Full Mars” conditions. Diurnal cycles are indicated by grey-colored areas showing nighttime (solar lamp switched off) and bluish areas showing daytime (lamp switched on).

Hydrated biofilms of *Chroococcidiopsis* sp. CCMEE 010 were exposed to Mars-like conditions for 3 days. Two VL biofilms and two FRL biofilms were exposed to “Full Mars” conditions, while two VL biofilms and two FRL biofilms were exposed to “Dark Mars” conditions (Figure 1A–C). Figure 2 shows the temperature and humidity trends over a 3-day simulation of night and day phases under “Full Mars” conditions. The pressure inside the Experiment Chamber was constant at about 600 Pa. The UV lamp was active for 16 h and switched off for 8 h daily to simulate the mid-latitude summer solar diurnal cycle, resulting in a total of 48 h of irradiation and a final cumulative dose of 2500 kJ/m^2^. The temperature varied diurnally between +15 °C (day) and −55 °C (night), whereas the relative humidity (with respect to ice) ranged between approximately 0.1% (day) and 100% (night). Under the “Dark Mars” conditions, UV irradiance and PAR light were absent, and the biofilms were exposed to Mars-like atmosphere, humidity, and temperature.

### 3.2. Photosynthetic Activity in Biofilms During Mars Simulation

**Figure 3:**
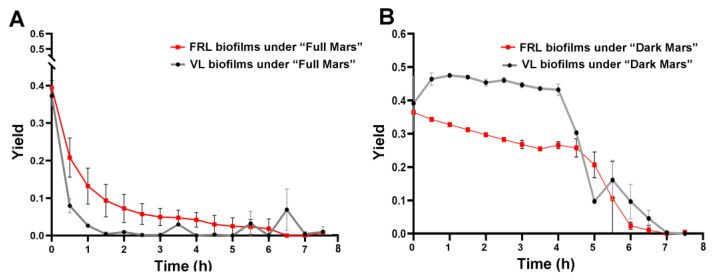
Maximum quantum efficiency of photosystem II during 3 days of Mars simulation. FRL and VL biofilms during “Full-Mars” exposure (**A**). FRL and VL biofilms during “Dark-Mars” exposure (**B**). Values are presented as means with standard errors represented by vertical bars.

Figure 3 shows the maximum quantum yield of the PSII, an indicator of the photosynthetic activity of the VL and FRL biofilms of *Chroococcidiopsis* sp. CCMEE 010 during the 3-day simulation. Under both “Full Mars” and “Dark Mars” conditions, the yield value decreased to undetectable values within 8 h of simulation (Figure 3A,B). Under “Full Mars” conditions, the reduction was sharper in the VL biofilms than in the FRL biofilms, and it occurred after 1 h of exposure (Figure 3A). Under “Dark Mars” conditions, the yield reduction rate differed between the VL and FRL biofilms; in the VL biofilms, it decreased sharply after 4 h of simulation, while a slow reduction occurred in the FRL biofilms until undetectable values were reached after 8 h of simulation (Figure 3B).

### 3.3. Visual Inspection of Biofilms Exposed to Mars Simulation

**Figure 4:**
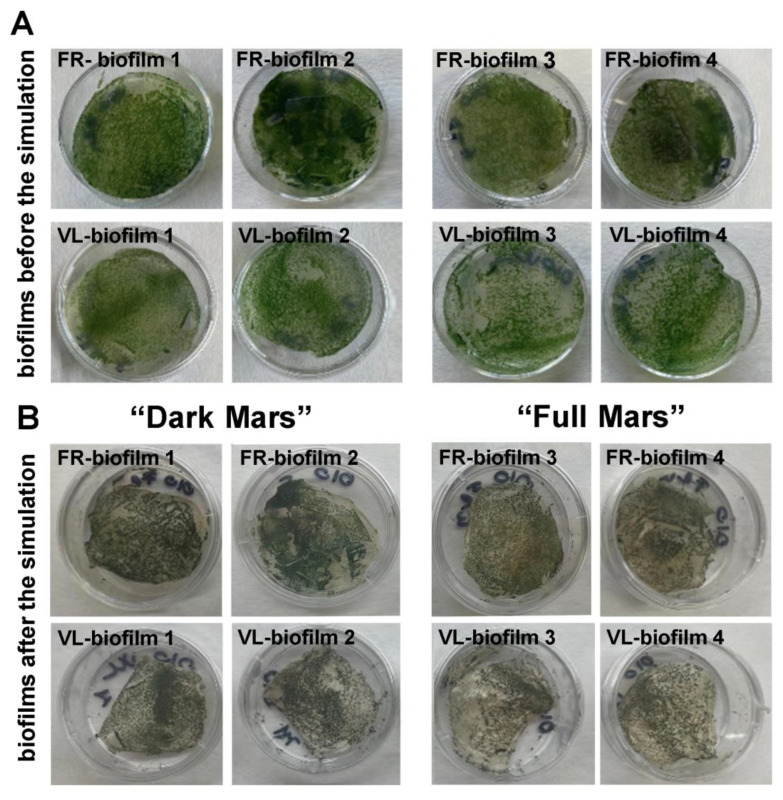
Visual inspection of *Chroococcidiopsis* sp. 010 biofilms. FRL and VL biofilms before (**A**) and after 3 days of Mars simulation (**B**).

All biofilms exposed to the Mars simulation were visually inspected. Before their exposure to Mars-like conditions, the hydrated *Chroococcidiopsis* sp. CCMEE 010 biofilms showed a typical green coloration regardless of whether they developed under VL or FRL conditions (Figure 4A).

After the 3-day Mars simulation, the biofilms were removed from the simulation chamber, and a visual inspection revealed that they underwent desiccation (Figure 4B). In addition, the dried biofilms showed a greyish-green coloration that was slightly more evident in the biofilms exposed to “Full Mars” conditions compared to those exposed to “Dark Mars” conditions and, therefore, not exposed to UV irradiation or PAR light (Figure 4B).

### 3.4. Photosynthetic Pigments in Biofilms Exposed to Mars Simulation

**Figure 5:**
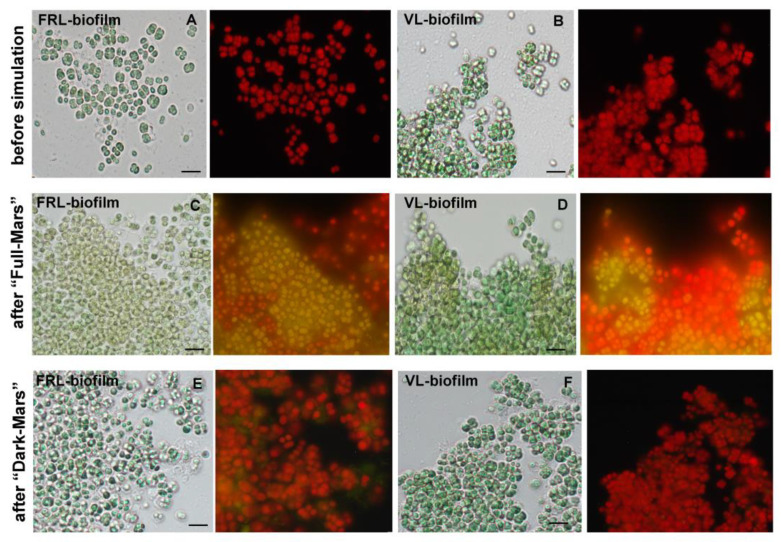
Epifluorescence microscopic images of *Chroococcidiopsis* sp. CCMEE 010 biofilms exposed to Mars-like conditions. VL and FRL biofilms before Mars simulation (**A**,**B**); VL and FRL biofilms exposed to “Full Mars” conditions (**C**,**D**); VL and FRL biofilms exposed to “Dark Mars” conditions (**E**,**F**). Scale bars indicate 20 µm.

Before their exposure to Mars-like conditions, the hydrated *Chroococcidiopsis* sp. CCMEE 010 biofilms exhibited a typical green color and red autofluorescence under bright-field and epifluorescence microscopy (Figure 5A,B). After the 3-day Mars simulation, the VL and FRL biofilms exposed to the “Full Mars” conditions both exhibited bleached cells with reduced photosynthetic pigment autofluorescence and cells retaining red autofluorescence (Figure 5C,D). On the other hand, an intense red autofluorescence occurred in the VL and FRL biofilms exposed to “Dark Mars” conditions (Figure 5E,F).

### 3.5. Cell Viability of Biofilms Exposed to Mars Simulation

**Figure 6:**
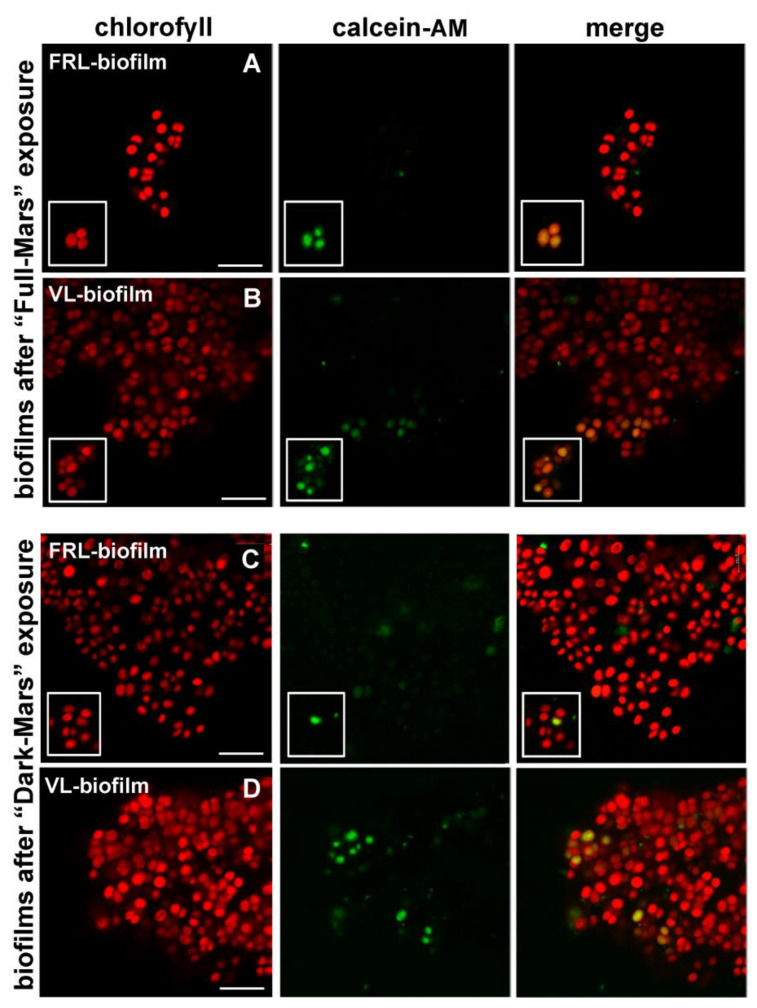
CLSM images of *Chroococcidiopsis* sp. CCMEE 010 biofilms stained with calcein-AM after Mars-like simulation. FRL biofilms (**A**) and VL biofilms (**B**) exposed to “Full Mars” conditions with pigment autofluorescence; viable cells with calcein signal. FRL biofilms (**C**) and VL biofilms (**D**) exposed to “Dark Mars” conditions with pigment autofluorescence; viable cells with calcein signal. Inserts with details of viable cells according to calcein-AM assay. Scale bars indicate 30 µm.

After 3 days of Mars-like conditions, cell viability was determined using an esterase activity test based on calcein-AM, a non-fluorescent, cell-permeant staining agent that produces a fluorescent derivate when hydrolyzed by cellular esterases. Under “Full Mars” conditions, in the VL biofilms, living cells showed a green calcein signal and red autofluorescence in the photosynthetic pigments (Figure 6B), while in the FRL biofilms, only a few cells were scored after extensive observation under the microscope (Figure 6A), and thus considered close to 0%. Under “Dark Mars” conditions, living cells were found in both the VL and FRL biofilms (Figure 6C,D). The percentage of survival in the VL biofilms under “Full Mars” conditions corresponded to about the 12% of the cell population, while in VL and FRL biofilms under “Dark Mars” conditions, the percent survival was about 80% and 35% of the cell population, respectively. In unexposed VL and FRL biofilms, living cells comprised about 90% of the cell pollution.

## 4. Discussion

The aim of this work was to extend the current knowledge of the response of the desert cyanobacterium *Chroococcidiopsis* to Mars-like conditions and gain insights into the possibility of a phototrophic life-form colonizing near-surface niches during the late-stage evolution of habitable conditions on Mars. Therefore, the desert isolate *Chroococcidiopsis* sp. CCMEE 010, which is capable of FaRLiP, was used as an experimental strain to obtain VL- and FRL-grown biofilms that were exposed to a Mars simulation in the metabolically active state. Indeed, biofilm formation represents a survival strategy since the shift from a planktonic free-living state to a sessile lifestyle provides an increased resistance to various environmental stressors that is due, among other factors, to abundant extracellular polymeric substances [30]. In the context of the Biofilm Organisms Surfing Space experiment, desert *Chroococcidiopsis* strains in the form of dried biofilms were exposed to Mars-like conditions in low Earth orbit, and their enhanced survival compared to their planktonic counterparts was noted [23]. In particular, dried biofilms of *Chroococcidiopsis* sp. CCMEE 029 survived 300 kJ/m^2^ of UV radiation (200–400 nm) during exposure in low Earth orbit as opposed to dried monolayers that survived 15 kJ/m^2^ of a Mars-like flux in a laboratory simulation [31].

Hydrated and metabolically active *Chroococcidiopsis* sp. CCMEE 010 biofilms developed under VL and FRL could withstand 3 days of exposure to a laboratory simulation of Mars conditions, although with differences in the survival percentage and photoinhibition rate. At the beginning of the Mars simulation, the VL and FRL biofilms showed a maximum quantum yield of about 0.4, which is in agreement with the value reported for the desert strain *Chroococcidiopsis* sp. CCMEE 029 under optimal growth conditions [32]. However, in the first hours of the 3-day simulation, the photosynthetic efficiency dropped to undetectable values with a different time-dependent rate under “Full Mars” conditions compared to “Dark Mars” conditions.

When exposed to “Full Mars” conditions, the VL biofilms showed a maximum quantum efficiency that dropped sharply within 1 h of exposure, whereas for the FRL biofilms, a slow reduction occurred until undetectable values were reached after 8 h of exposure. Even though the MiniPAM coupled to the Mars Simulation Chamber determined the efficiency of the photosystem II and not that of photosystem I, it is considered a useful tool to reveal perturbations in the photosynthetic process, since photosystem II is crucial in the photosynthetic electron transport, as it governs linear electron flow by splitting water and providing electrons to photosystem I [28].

The underlying mechanism of resilience of the FRL biofilms warrants investigation, for instance, through an integrated transcriptomic or proteomic analysis. At present, it is reasonable to speculate that, since only VL (PAR illumination) was available during the Mars simulation, the FRL biofilms might have entered a transition process to re-acclimate to the VL condition [33]. Whether this transition rendered the photosystems less susceptible to photoinhibition remains unclear, although it could be possible that the remodeling of the FRL photosystem might have attenuated indirect damage induced by UV radiation through the production of reactive oxygen species.

On the other hand, when exposed to “Dark Mars” conditions, the VL and FRL biofilms underwent a slow reduction in maximum quantum efficiency, although during the first 5 h, higher values occurred in the VL biofilms compared to the FRL biofilms. This suggests that UV irradiation was the primary cause of photoinhibition. Indeed, under “Full Mars” conditions, the biofilms received a cumulative UV radiation dose of 2500 kJ/m^2^, whereas under “Dark Mars” conditions, they experienced a Mars-like atmosphere, humidity, and temperature. Notably, during the 3-day simulation, photosynthetic activity was not restored in the VL or FRL biofilms after the initial drop, although it may be speculated that a longer simulation (lasting days) could yield other results.

A different reduction in the photochemical yield under “Full Mars” and “Dark Mars” conditions was reported for the lichen *Xanthoria parietina*, which showed decreases of 54% and 42%, respectively, after the first 4 h of the Mars simulation [27]. The photochemical yield then increased again, reaching the highest values at night, when the relative humidity reached its maximum, and the lowest values during the day, with minimum relative humidity values [27]. On the contrary, the lichen *Pleopsidium chlorophanum* showed a drop in the photochemical yield in a Mars simulation under a final cumulative UV radiation dose of 6344 kJ/m^2^, and it was unclear if the algal symbiont was actively photosynthesizing [34]. On the contrary, increased photosynthetic activity occurred in a lichen exposed to a final cumulative UV radiation dose of 269 kJ/m^2^ over a period of 34 days, representing the low dose of radiation that would be encountered in near-surface, semi-protected loci, e.g., fissures and cracks in rocks [34].

Even though the “Mars Dark” conditions were less detrimental to the *Chroococcidiopsis* biofilms due to the lack of UV-induced damage, the low pressures, high CO_2_ concentrations, and extreme temperature fluctuations they were exposed to also impaired the photosynthetic process. Low pressures limit microbial metabolism [35]; although lichens can maintain photosynthetic activity in a Mars-like atmosphere [27,34], such a capacity has not been reported for cyanobacteria [36].

At the beginning of the Mars simulation, the humidity and temperature values kept the *Chroococcidiopsis* biofilms in the hydrated state. The humidity and temperature values then dropped, causing biofilm desiccation, while the pressure remained constant at 600 Pa. However, since living cells were noted within the dried biofilms, it may be possible that they survived the diurnal cycle of humidity and temperature by entering a dried, dormant state.

A post-exposure analysis of the *Chroococcidiopsis* biofilms revealed the presence of bleached photosynthetic pigments and cells that retained pigment autofluorescence. This is not unexpected since the top-layer cells might have provided protection from UV radiation during Mars simulation, even though the permanence of photosynthetic pigment autofluorescence alone is not an indicator of viability. However, living cells were found in both VL and FRL biofilms exposed to “Dark Mars” and Full Mars” conditions, as revealed by the calcein-AM viability test. Living cells showed a green calcein signal in agreement with the ability of this nonfluorescent vital dye to cross the cellular membrane and be converted into a fluorescent dye by cytosolic esterases [37]. On the contrary, dead cells lacked the green calcein signal but maintained the red photosynthetic pigment autofluorescence. The fact that, after their exposure to “Full Mars” conditions, survival of about 12% was noted in the in VL biofilms while only a few live cells were observed in the FRL biofilms suggests that biofilms pre-acclimated to FRL were more sensitive to UV irradiation. Moreover, under “Dark Mars” conditions, the survival of the FRL biofilms was reduced by more than a half compared to the VL biofilms. During the Mars simulation, the FRL biofilms might have initiated a VL acclimation process, and this prioritization might have impaired other biosynthetic pathways needed to face the desiccation process. On the contrary, under the Mars simulation conditions, the VL biofilms might have established adaptation mechanisms, resulting in enhanced survival. Nevertheless, it is remarkable that the *Chroococcidiopsis* biofilms survived a final cumulative UV radiation dose of 2500 kJ/m^2^, which was one magnitude higher than that experienced by dried biofilms exposed to a Mars-like UV lux in low Earth orbit during the BOSS space mission due to the presence of 0.1% neutral density filters on the EXPOSE-R2 facility [23].

## 5. Conclusions

Biofilms of *Chroococcidiopsis* sp. CCMEE 010 were exposed for the first time in a metabolically active state to laboratory simulations of Mars conditions. The photosynthetic activity dropped in both VL- and FRL-grown biofilms, which entered a dried, dormant state with the top-layer cells shielding the bottom-layer cells from UV radiation. This enabled the reconstruction of possible events leading to a late extinction of land communities on Mars [7]. The ability to drive photosynthesis using low-energy photons that reach rocky substrates highlights the possibility that near-surface, semi-protected niches could have been colonized by phototrophs during the late-stage evolution of habitability on Mars. The endurance of cyanobacterial cells encased in a biofilm structure in Mars-like conditions expands the boundary conditions that life cannot face as a single cell. Moreover, the biofilms’ UV resistance suggests that, during the loss of surface habitability on Mars, putative microbial life-forms might have survived surface conditions before taking refuge in near-surface protected niches, such as fissures or cracks in rocks [9]. Moreover, biofilms’ UV resistance has implications for the lithopanspermia (i.e., the interplanetary transport of microbial passengers inside rocks) by contributing to microbial survival upon arrival after the space transfer, with implications for the origin of life on Earth and within the solar system [38]. The resilience of *Chroococcidiopsis* sp. CCMEE 010 biofilms under “Full Mars” conditions aligns with the observation that the lack of an ozone shield is not, in and of itself, a limit to land colonization by phototrophs, even in the absence of other UV absorbers in the atmosphere apart from carbon dioxide [8]. This is relevant for the potential colonization of land on Archean Earth that would have been prevented by the lack of ozone, unless phototrophs, as the ‘worst-case organism’ since they depend on light, evolved UV resistance [39]. When applied to the definition of the habitability of exoplanets orbiting M-stars, the UV resilience of *Chroococcidiopsis* biofilms suggests that, even in the worst-case assumptions of UV radiation, early land masses of exoplanets in the habitable zone (i.e., orbiting at a distance from the star at which liquid water could exist on its surface) could be colonized by primitive phototrophs.

## Figures and Tables

**Table 1:** Environmental conditions inside the Mars simulation chamber.

Experimental Parameters	“Full Mars”	“Dark Mars”
Atmosphere	95% CO_2_, 4% N_2_, 1% O_2_	95% CO_2_, 4% N_2_, 1% O_2_
Pressure	600 Pa	600 Pa
PAR (400–700 nm)	3 W/m^2^	/
UV (200–400 nm)	14 W/m^2^	/
Humidity	0% (day)	0%
	75% (night)	75% (night)
Temperature	+15 °C (day 16 h)	+15 °C (day 16 h)
	−50 °C (night 8 h)	−50 °C (night 8 h)

## Data Availability

Dataset available on request from the authors.
